# Supplementary material for: A randomised controlled trial to test the effects of fish aggregating devices (FADs) and SBC activities promoting fish consumption in Timor-Leste: A study protocol
Source: PLoS One. 2022 Jul 8;17(7):e0269221. doi: 10.1371/journal.pone.0269221 (PMC9269458; doi:10.1371/journal.pone.0269221)
Supplement: S2 Appendix — (PDF) [file pone.0269221.s002.pdf]

MINISTÉRIO DA

SAÚDE

Instituto Nasional de Saúde
Diretora Ezecutivo

No Ref.: 1934MS-INS/DE/ /XII/2020
Dili, December 2th 2020

APROVAL LETTER

Alex Tilley PhD (Principal Investigator)
Ministerio·Agrikultura no Pesca TL

Caro Alex

Título do projeto: The effects of fish aggregating devices and nutrition SBCC on fish consumption in Timor Leste: A randomized controlled trial.
Obrigado por enviar o projeto de pesquisa acima para avaliação ética. Este projeto foi aprovado pelo Comitê Ética/Técnico do Insituto Nasional de Saúde numa reunião realizada em 23 de Novembro de 2020.

Tenho o prazer de informar que o Comitê Ética/Técnico do Insituto Nasional de Saúde concedeu aprovação ética deste projeto de pesquisa.
Por favor, note que, se outros sitios forem contratados antes do início do projeto de pesquisa ou durante o projeto de pesquisa, o investigador principal ou seja coordenador é obrigado a notificar ao Comitê Ética/Técnico do Insituto Nasional de Saúde. A notificação de sitios retirados também deve ser fornecida ao Comitê Ética/Técnico em certo tempo.

Os documentos aprovados incluem:
1 . Formulário de inscrição ou Formatu Aplicação (original)
2 . A eletrônica e cópias dos documentos de pesquisa, tais como formulários de consentimento, declaração de confidencialidade, questionários semi entrevistas (versão Portugues e Tetun).

Esta aprovação é por um período de quatro (4) meses. É necessário um relatório de progresso do projeto ANUAL / FINAL em ou antes de 24 de Março de 2021.
APROVAÇÃO ÉTICA SUJEITADO por seguintes condições preenchidas:

1. O Investigador Principal informará imediatamente qualquer coisa que possa rever a aprovação ética do projeto.
2. O investigador principal notificará o Comitê Ética/Técnico de qualquer evento que exija uma modificação no protocolo ou outro documento do projeto e submeta as alterações necessárias e de acordo com as instruções fornecidas pelo Comitê Ética/Técnico do Instituto Nasiona de Saúde.
3. O Investigador Principal ou seja coordenador enviará qualquer relatório necessário relacionado à segurança do participante da pesquisa (ou seja, desvio do protocolo, violações do protocolo) de acordo com a política e os procedimentos do comitê de ética em pesquisa no Instituto Nasiona de Saúde.

4. O Principal investigador em coordenação reportará ao Comité Ética/Ténicnico, anualmente no formato especificado e notificará o projeto for concluído em todos os sitios.
5. O Principal investigador em coordenação notificará ao Comité Ética/Ténicnico se o projeto for descontinuado no local participante antes da data de conclusão esperada, com o motivo fornecido.
6. O Principal investigador em coordenação notificará ao Comité Ética/Ténicnico de qualquer plano para prolongar a duração do prazo de aprovação do período de aprovação listado acima e enviará qualquer documentação necessária associada.
7. O Principal investigador ou seja coordenador notificará ao Comité Ética/Ténicnico de sua incapacidade de continuar como PI coordenador, incluindo o nome e informações de contato para uma substituição.
8. A conduta segura e ética deste projeto é inteiramente da responsabilidade dos investigadores e suas instituições.
9. O pesquisador deve informar imediatamente qualquer coisa que possa afetar a aceitação ética contínua do projeto, incluindo:
   - Efeitos adversos do projeto sobre assunto e etapas tomadas para lidar com estes; outros eventos imprevistos.
   - Novas informações que podem invalidar a integridade ética do estudo; e
   - Propor mudanças no projeto.
10. A aprovação por mais doze meses será concedida se o Comité Ética/Ténicnico estiver convencido de que o projeto foi consistido no protocolo original.
11. Confidencialidade os participantes da pesquisa devem ser mantidos em todos os momentos conforme necessário por lei.
12. A ficha informativa do paciente e o formulário de consentimento devem ser impressos no cabeçalho do sitio com detalhes de contato completo.
13. A folha de informações do paciente deve fornecer um breve resumo da atividade de pesquisa incluindo, riscos e benefícios, opções de retirada, detalhes de contato do pesquisador e também deve indicar que o Secretário de Comité Ética/Ténicnico pode ser contatado (Telefone e E-mail) para informações sobre políticas, direito de participante, preocupação ou queixa sobre a conduta ética do estudo.

Esta carta constitui apenas aprovação ética.

Se houver alguma dúvida sobre a consideração de seu projeto pelo Comité Ética/Técnico, entre em contato com o Departamento Peskiza no Instituto Nasional de Saúde com o número telefone 3310611. O Comité Ética/Ténicnico do INS deseja-lhe todo o sucesso em sua pesquisa.
Se houver algumas dúvidas ou seja a revisão do seu projeto, entre em contato com o Departamento Pesquiza no Instituto Nasional de Saúde.

Com os melhores cumprimentos,

Domingas da C. Pereira, L.E.
Diretora Executiva e Presidente da
Conselho Administrativo do INS
