# Supplementary material for: A randomised controlled trial to test the effects of fish aggregating devices (FADs) and SBC activities promoting fish consumption in Timor-Leste: A study protocol
Source: PLoS One. 2022 Jul 8;17(7):e0269221. doi: 10.1371/journal.pone.0269221 (PMC9269458; doi:10.1371/journal.pone.0269221)
Supplement: Attachment — Submitted filename: Response to reviewers.docx [file pone.0269221.s003.docx]

Response to reviewers

We thank the reviewers for their thoughtful comments – they have helped to make the manuscript much stronger. Please see our responses to each comment below.

**Editor:**

1. Please ensure that your manuscript meets PLOS ONE's style requirements, including those for file naming. The PLOS ONE style templates can be found at

<https://journals.plos.org/plosone/s/file?id=wjVg/PLOSOne_formatting_sample_main_body.pdf> and <https://journals.plos.org/plosone/s/file?id=ba62/PLOSOne_formatting_sample_title_authors_affiliations.pdf>.

**Response**: The manuscript has been formatted accordingly and files have been renamed where necessary according to PLOS One file naming conventions.

2. We note that you have stated that you will provide repository information for your data at acceptance. Should your manuscript be accepted for publication, we will hold it until you provide the relevant accession numbers or DOIs necessary to access your data. If you wish to make changes to your Data Availability statement, please describe these changes in your cover letter and we will update your Data Availability statement to reflect the information you provide.

**Response**: The data availability statement is unchanged

3. Please include captions for your Supporting Information files at the end of your manuscript, and update any in-text citations to match accordingly. Please see our Supporting Information guidelines for more information: <http://journals.plos.org/plosone/s/supporting-information.>

**Response**: The Supporting Information has been deemed unnecessary to include, as it was the consent form, which is standard across studies. It can be supplied upon request

4. We note that Figure 3 in your submission contain [map/satellite] images which may be copyrighted. All PLOS content is published under the Creative Commons Attribution License (CC BY 4.0), which means that the manuscript, images, and Supporting Information files will be freely available online, and any third party is permitted to access, download, copy, distribute, and use these materials in any way, even commercially, with proper attribution. For these reasons, we cannot publish previously copyrighted maps or satellite images created using proprietary data, such as Google software (Google Maps, Street View, and Earth). For more information, see our copyright guidelines: <http://journals.plos.org/plosone/s/licenses-and-copyright>.

**Response**: Fig 3 (now Fig 4) was drawn as a vector image by AT so does not require copyright information.

**Reviewer 1:**

(1) The study protocol is not registered in a public accessible database approved by the WHO or ICMJE.

**Response**: We have registered the protocol with clinicaltrials.gov, which is an approved database by the WHO. This database is publicly accessible and searchable.

The link to the clinialtrials.gov registry can be found here (NCT04729829) https://clinicaltrials.gov/ct2/show/NCT04729829?term=NCT04729829&draw=2&rank=1

(2) The name of the registry and the trial or study registration number is not included in the abstract.

**Response**: This has been added.

(3) A SPIRIT schedule of enrollment, interventions, and assessments is not included as the manuscript’s Figure 1.

**Response:** Figures 1 and 2 include all of the information about enrolment, interventions, and assessments. We also provide a clear description of the interventions in Table 1.

(4) In general, I would suggest to follow the SPIRIT recommendations step-by-step rigorously throughout the manuscript.

**Response**: We used the CONSORT checklist, which is used for reporting randomized controlled trials, and is a standard, detailed tool.

(5) Moreover, including a data management plan and statistical analysis plan should be added as supporting information.

**Response:** We have clearly laid out the data management plan and statistical analysis plan in the main text (Sections 2.7 and 2.8). In the mentioned sections, we have explained how variables will be measured, the descriptive analysis, econometrics approach, and mediation analysis. Following the CONSORT checklist tool (S1 Appendix), we have included all of the required information in our manuscript and the corresponding page number.

**Reviewer 2:**

Line 106: the sentence seems incomplete

**Response**: Thus was a piece of text was accidentally left during the editing process. It has been deleted.

Line 90: Although the trial design and sample size and sampling procedure are clear, the numbers (n = ) indicated in the CONSORT flow chart are confusing. How many inland villages are there? 24 or 12? How many consumers? 700 or 720?

**Response**: We agree that this is confusing and we have edited the diagram. Please see the updated version in Figure 1.

Line 176: What type of FADs are you considering deploying? The use of wood, concrete or plastic FADs could improve or affect the trial throughout the year. A description of the aggregating devices to be used would be very useful.

**Response**: In the *Trial design* section, we have added a new description and diagram of the FAD design (Fig 3) and linked to the publication that describe and test the FADs and their deployment methods.
