# Supplementary material for: A randomised controlled trial to test the effects of fish aggregating devices (FADs) and SBC activities promoting fish consumption in Timor-Leste: A study protocol
Source: PLoS One. 2022 Jul 8;17(7):e0269221. doi: 10.1371/journal.pone.0269221 (PMC9269458; doi:10.1371/journal.pone.0269221)
Supplement: Attachment — Submitted filename: Response to reviewers - v3.docx [file pone.0269221.s004.docx]

December 18, 2021

We thank the reviewers for their thoughtful suggestions, and the manuscript has been improved as a result. Please see our responses below.

Reviewer comment 1:

The manuscript should describe the methods in sufficient detail to prevent undisclosed flexibility in the experimental procedure or analysis pipeline, including sufficient outcome-neutral conditions (e.g., necessary controls, absence of floor or ceiling effects) to test the proposed hypotheses and a statistical power analysis where applicable. As there may be aspects of the methodology and analysis which can only be refined once the work is undertaken, authors should outline potential assumptions and explicitly describe what aspects of the proposed analyses, if any, are exploratory.

Response: We have specified that control variables to be used in the main estimation equation (1) will come from panel B of Table 2. This helps to minimize the tendency for cherry-picking controls. The way variables are measured could affect the estimated results. Therefore, the methods sections clearly explains how each variable will be measured. We also recognize that the stable unit treatment value assumption (SUTVA) might be violated if other organizations working in the study sites implement interventions similar to those proposed in the study. This is especially problematic if the control group is targeted because of the feeling that they have been left out of a potentially beneficial intervention. We have made efforts to control for this potential problem by collecting data about all organizations working in the study site and the interventions implemented (current and planned). In addition to the randomization, which helps to ensure both treatment and control group participants are equally exposed to the interventions of such organizations at baseline, we discussed with partner organizations and understood their planned future interventions and found that they do not relate to the interventions implemented in our study. Furthermore, we will collaborate with Mercy Corps as our implementation partner to ensure alignment in project interventions and to avoid parallel interventions being targeted at the same respondents by different organizations. Changes have been made in the methodology section accordingly.

Reviewer comment 2: I would like to ask you to organize the manuscript along SPIRIT and address all aspects of the checklist.

Response: This manuscript has been updated and revised accordingly. Please see the supplementary SPIRIT checklist attached.
