# Supplementary material for: A randomised controlled trial to test the effects of fish aggregating devices (FADs) and SBC activities promoting fish consumption in Timor-Leste: A study protocol
Source: PLoS One. 2022 Jul 8;17(7):e0269221. doi: 10.1371/journal.pone.0269221 (PMC9269458; doi:10.1371/journal.pone.0269221)
Supplement: Attachment — Submitted filename: Response to Reviewers.docx [file pone.0269221.s005.docx]

There was only one comment from Reviewer #1:

“**Please add where all data underlying the findings will be made available when the study is complete (see 4.). All my other comments have been satisfactorily addressed**.”

In response, we have added the following text to clarify where the data will be made available online (lines 258-263):

*Identifiable survey data will be archived and accessible to only the research team and those approved by the entire research team. Anonymised data from the study baseline survey will be published on the Harvard Dataverse (*[*https://doi.org/10.7910/DVN/VZJYIZ*](https://doi.org/10.7910/DVN/VZJYIZ)*) to facilitate future research such as pooled analysis with other studies for purposes of meta-analysis or multi-site analysis. Anonymous fisheries landings data are publicly available on Harvard Dataverse (*[*https://dataverse.harvard.edu/dataverse/peskas*](https://dataverse.harvard.edu/dataverse/peskas)*) and updated automatically every month.*
